# Spongiotic gingival hyperplasia: a systematic review of histopathologic features

**DOI:** 10.3389/froh.2026.1780111

**Published:** 2026-03-26

**Authors:** Lama Alabdulaaly, Asma Almazyad

**Affiliations:** 1 Maxillofacial Surgery and Diagnostic Sciences Department, College of Dentistry, King Saud bin Abdulaziz University for Health Sciences, Riyadh, Saudi Arabia; 2 Dental Services Department, Ministry of the National Guard Health Affairs, Riyadh, Saudi Arabia; 3 King Abdullah International Medical Research Center, Riyadh, Saudi Arabia; 4 Department of Oral Medicine, Infection, and Immunity, Harvard School of Dental Medicine, Boston, MA, United States

**Keywords:** biopsy, gingiva, gingival hyperplasia, gingivitis, oral pathology

## Abstract

**Objectives:**

Spongiotic gingival hyperplasia (SGH) is a rare inflammatory gingival condition that exhibits characteristic histopathologic features. The aim of this study was to systematically summarize the reported histopathologic features of SGH.

**Study design:**

A systematic review of SGH literature following the Preferred Reporting Items for Systematic Reviews and Meta-analyses (PRISMA) statement was performed. PubMed, Wiley, and Web of Science were searched for publications of biopsy-proven SGH cases.

**Results:**

In total, 25 articles were included (289 patients). SGH occurred mostly in the first two decades with equal sex distribution. Multifocal lesions were reported in 13.5% of patients. A predilection for the maxillary anterior gingiva was noted. The majority of lesions were covered by non-keratinized epithelium (73.6%). Acanthosis/hyperplasia was noted in 88.4% of cases, and neutrophilic transmigration was noted 62.0% of cases. Highly vascular lamina propria was a common feature. Moreover, 99% of cases stained for cytokeratin 19 demonstrated positivity throughout the full epithelial thickness. Excision was the most common treatment, and the majority of cases resolved.

**Conclusions:**

SGH is a reactive gingival pathology that primarily occurs in adolescents. The histopathologic features of SGH consistently reported were hyperplastic non-keratinized stratified squamous epithelium with spongiosis and prominent vascular stroma. Surgical excision was curative in the majority of cases.

**Systematic Review Registration:**

PROSPERO CRD420251170524.

## Introduction

**Figure 1:**
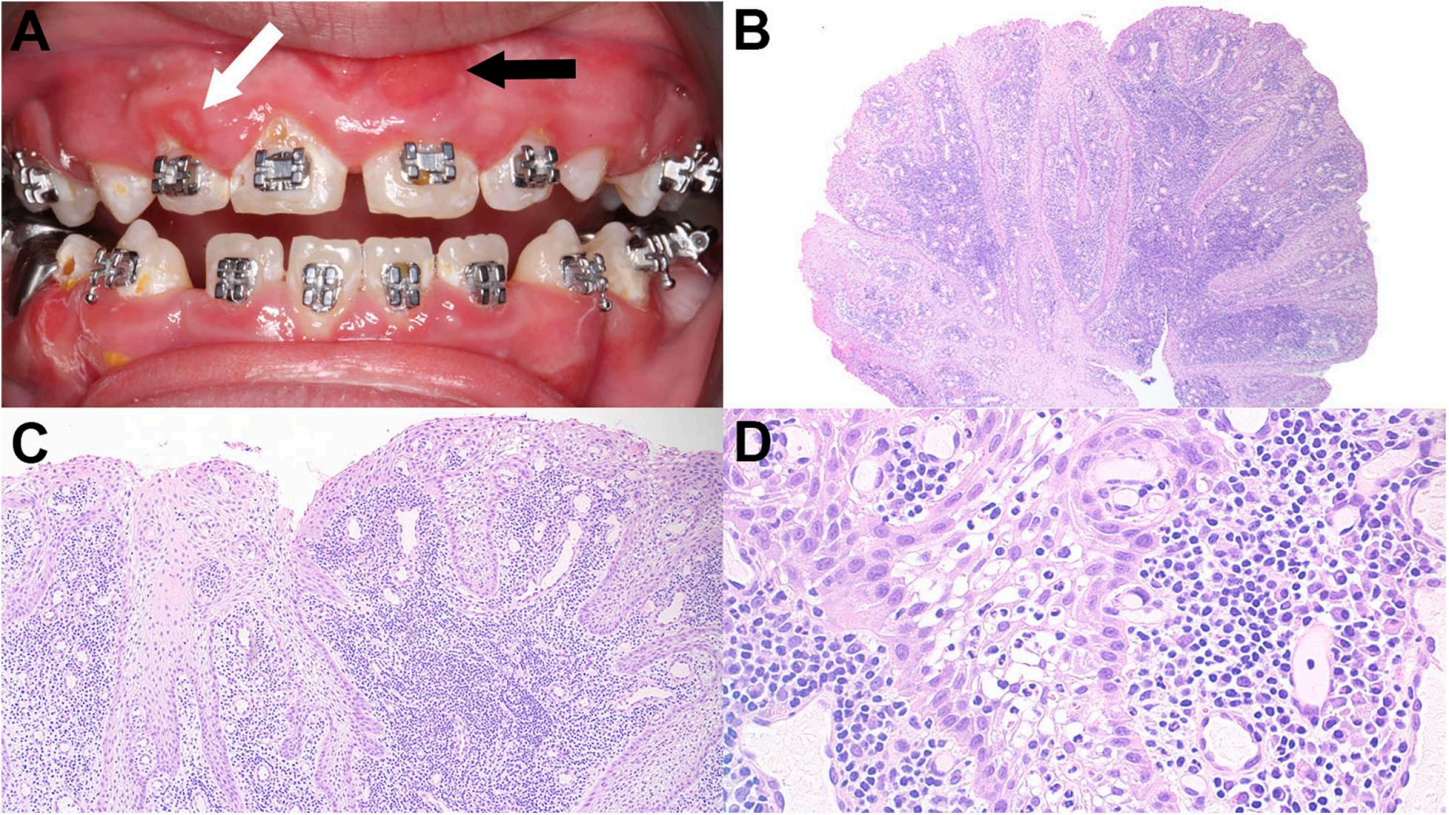
Clinical and typical histopathologic appearance of spongiotic gingival hyperplasia. **(A)** Diffuse gingival enlargement with multiple well-delineated erythematous plaques (arrows). The black arrow indicates the biopsy site. Courtesy of Dr. Ghada Alzamil, Riyadh, Saudi Arabia. **(B)** Low-power view of the erythematous gingival lesion depicted in Figure 1A. **(C)** The lesion is covered by non-keratinized stratified squamous epithelium. The epithelium is hyperplastic and demonstrates thinning of the suprapapillary plate. **(D)** Prominent spongiosis and neutrophilic transmigration. The lamina propria is vascular and exhibits acute and chronic inflammation.

Spongiotic gingival hyperplasia (SGH), formerly known as localized juvenile spongiotic gingivitis/gingival hyperplasia (1, 2), is an uncommon gingival condition with unknown etiology (3). SGH characteristically presents as an erythematous raised plaque, often with a papillary or granular surface (Figure 1A) (2). SGH typically occurs on the gingiva, commonly on the maxillary gingiva (4), although extragingival involvement has been reported (2, 5). Reported cases demonstrated no sex predilection and spanned a wide age range, with a median age of 13 years (range: 7–73 years) (4). Early reports characterized SGH as a unifocal condition occurring primarily in younger individuals, leading to the use of the term *localized juvenile SGH*; however, subsequent studies documenting multifocal disease and involvement of older patients prompted the adoption of the more inclusive term SGH (3, 6).

Histopathologically, SGH exhibits exophytic papillary benign proliferation of non-keratinized stratified squamous epithelium (Figures 1B,C). Spongiosis, inflammatory cell transmigration, and a lamina propria rich in vasculature are characteristic features (Figure 1D) (6). These features may overlap with those of other inflammatory or reactive gingival lesions, posing diagnostic challenges in routine practice. The differential diagnosis of SGH includes dental biofilm-induced gingivitis, foreign body gingivitis, inflamed squamous papilloma, pyogenic granuloma, and gingival lesions associated with granulomatosis with polyangiitis (1–3, 7, 8). Dental biofilm-induced gingivitis can be distinguished from SGH by the presence of dental biofilm in proximity to the lesion. Furthermore, previous reports have demonstrated persistence of SGH lesions despite employing plaque control measures (9). Foreign body gingivitis is diagnosed following identification of a foreign body on biopsy specimens, which frequently elicits a granulomatous response (10). Although both squamous papilloma and SGH often demonstrate a papillary surface, SGH does not appear to be human papillomavirus (HPV)-driven, as shown previously (8). The primary histopathology of pyogenic granuloma is proliferation of endothelial cells and new blood vessel formation (11). Finally, gingival lesions associated with granulomatosis with polyangiitis exhibit diffuse non-necrotizing granulomatous inflammation, hemorrhage, and mixed inflammatory cell infiltrate in the setting of a multiorgan systemic disease (10). Therefore, clinicopathologic correlation is essential in identifying SGH lesions.

Management of SGH has most commonly involved surgical excision, while conservative approaches, including topical therapies and laser-based modalities, have also been described (12, 13). Occasional cases of spontaneous resolution have been reported (14). Approximately two-thirds of cases show no recurrence (65%–70%), whereas recurrence has been documented in a subset of patients (30%–35%), including lesions treated with complete surgical excision, underscoring the need for continued clinical surveillance (15).

Owing to the relatively recent characterization and rarity of this entity, the existing literature is largely limited to case reports and case series. Although a limited number of systematic reviews have addressed aspects of its clinical management, there remains a notable lack of focused synthesis of the histological and immunohistochemical features of SGH. Therefore, this systematic review aimed to comprehensively evaluate and report the histopathologic and immunohistochemical findings in SGH.

## Materials and methods

The Preferred Reporting Items for Systematic Reviews and Meta-analyses (PRISMA) statement was followed during the preparation of this systematic review. This study was registered with the International Prospective Register of Systematic Reviews (PROSPERO ID CRD420251170524).

### Information sources and search strategy

Three electronic databases, namely, PubMed, Wiley, and Web of Science, were searched on 31 October 2025, with no date restrictions. The search strategy included the following terms: *spongiotic gingival hyperplasia*, *localized juvenile spongiotic gingival hyperplasia*, *juvenile spongiotic gingivitis*, *localized juvenile spongiotic gingival inflammation*, and *localized spongiotic gingival hyperplasia*.

### Eligibility criteria

The following PICOS criteria were followed to determine eligibility for inclusion:

Population: Individuals with biopsy-proven SGH (previously termed “localized juvenile spongiotic gingival hyperplasia/gingivitis”).

Intervention: Biopsy.

Comparator: Not applicable.

Outcome: Results of histopathologic examination and ancillary testing.

Study design: Case reports, case series, retrospective studies, prospective studies, and randomized and non-randomized trials published in English.

### Exclusion criteria

Systematic reviews, narrative reviews, letters to the editors, and meeting abstracts were not considered in this systematic review. Publications that lacked a histopathologic diagnosis of SGH were excluded.

### Selection process

The two authors performed the selection process independently. The search results were downloaded to EndNote 20, and duplicates were removed using the “Find Duplicates” feature, followed by manual removal of any remaining duplicates. After removing duplicates, titles and abstracts were screened, and ineligible records were excluded. The remaining records were screened based on their full text. After full-text screening, the authors compared their results and resolved discrepancies through discussion.

### Data collection process and data items

One author (LA) extracted the data. The data collected from the articles included the number of patients, age or age group, sex, lesion duration, symptoms, associated factors such as orthodontic treatment, number of lesions per patient, lesion location, histopathologic features, immunohistochemical findings, ancillary testing, management, and outcomes. In this review, decades start from years ending in 0 and end on years ending in 9 (i.e., the second decade is from 10 to 19 years of age). In one publication (16), age was not reported, but age groups were reported as ≤10, 11–15, 16–20, and ≥61 years old; these patients were recorded in the closest decade in this review (i.e., the ≤10 years old group was recorded in the 0–9 years old group and so on). In case series that reported the mean age, all patients in such publications were considered to be of that age. The lesion duration and follow-up period were recorded only if reported as discrete numbers (ranges were excluded). Lesions were assumed to be single if multifocality was not reported. Lesions involving the gingiva of more than one tooth, but were contiguous, were considered single unless multifocality was reported in the article. Inflammation on histopathology was grouped into acute, chronic, and mixed based on the types of cells reported to be present. In one publication (17), immunohistochemical markers were reported to be positive, but the number of positive cases was not reported. Therefore, all 10 cases in the aforementioned study were considered positive for all the immunohistochemical markers performed in that study. For lesions that received multiple treatments, only the most recent treatment was considered here unless otherwise indicated. Lesions that did not recur were considered resolved lesions.

### Risk of bias assessment

The two authors independently conducted the risk-of-bias assessment using the Joanna Briggs Institute (JBI) Critical Appraisal Tool for case reports, case series, and case-control studies (18, 19). The risk of bias was determined based on the percentage of “yes” responses to the total number of questions. Articles that scored less than 50% were considered to have a high risk of bias, while low risk was assigned to articles that scored 70% or higher. Moderate risk was defined as scores between 50% and 69%. The authors had concordant risk scores for all but one article, and the discrepancy was resolved through discussion.

### Statistical analysis

The collected data were recorded in Microsoft Excel and reported descriptively. GraphPad Prism (version 10.2.3 for MacOS, Boston, MA, USA) was used for data visualization.

## Results

### Study selection and characteristics

**Figure 2:**
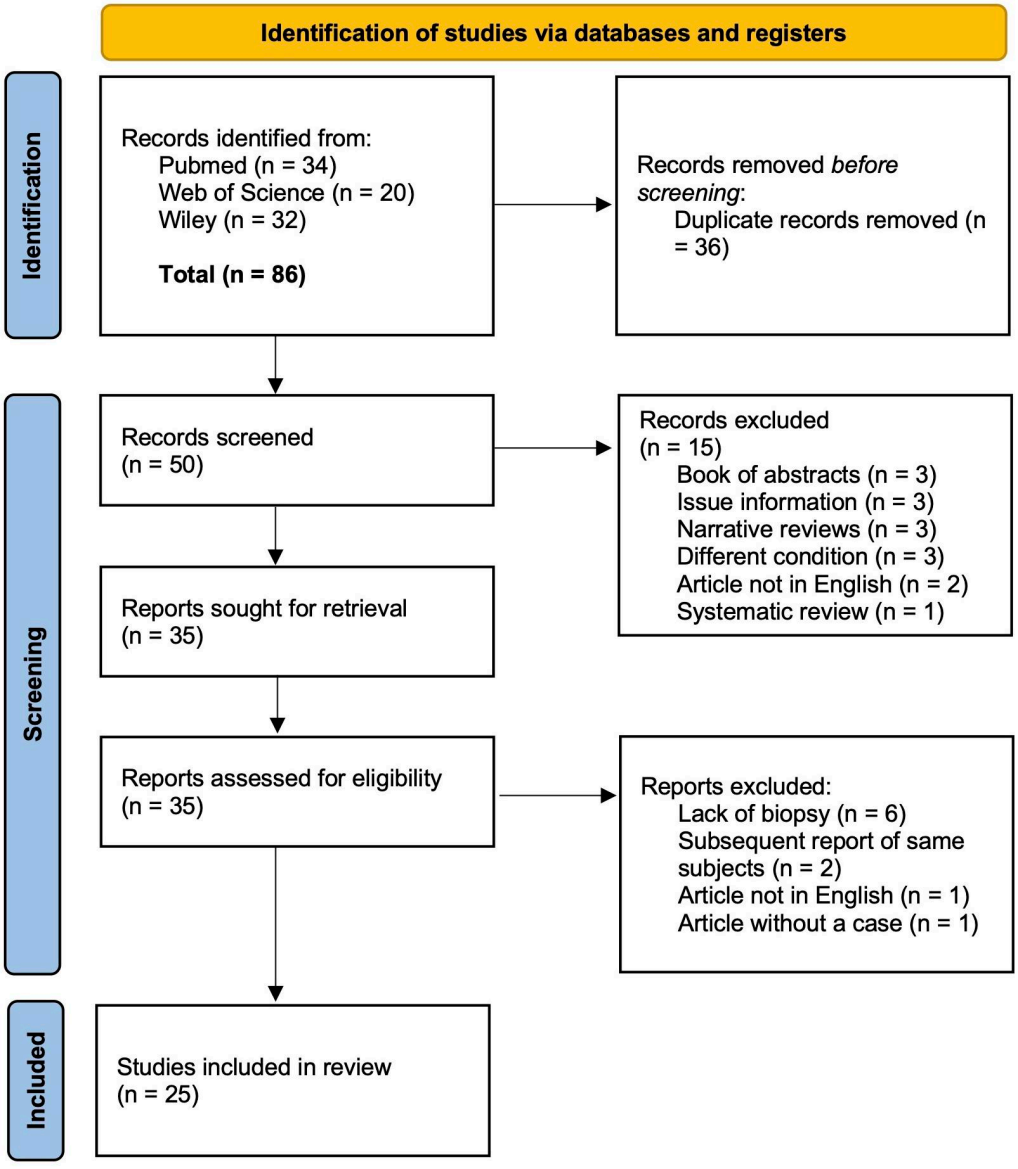
Preferred Reporting Items for Systematic Reviews and Meta-analyses (PRISMA) flowchart.

The search across the three databases yielded a total of 86 records. In total, 36 duplicate records were removed, and the remaining 50 records were screened based on titles and abstracts. After excluding 15 records, 35 full-text articles were screened. Following full-text screening, six articles were excluded due to a lack of histopathologic examinations (14, 20–24), two articles were excluded due to the subsequent inclusion of the patients in Theofilou et al. (6, 25, 26), one article was excluded because it was written in Chinese (27), and one record was excluded due to the absence of a case report (28). The number of studies reviewed in this systematic review was 25 articles (Figure 2). There were 14 case series (1–9, 16, 29–32), 10 case reports (12, 13, 33–40), and one case-control study (17).

### Demographics and clinical characteristics

**Figure 3:**
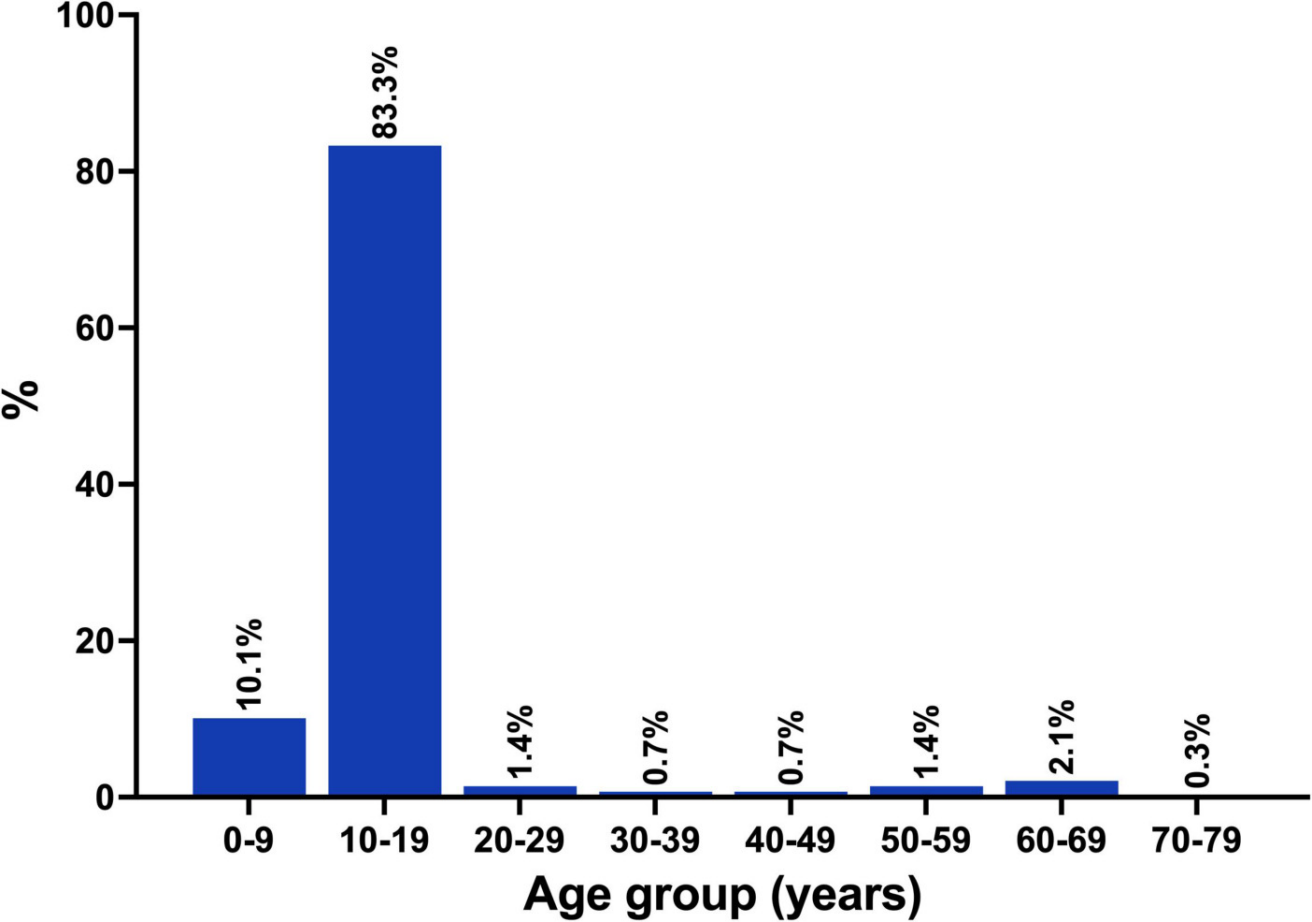
Age distribution.

A total of 292 biopsies from 289 patients were analyzed. The median age was 13 years (range: 3–78 years). The majority of patients (93.4%) were younger than 20 years of age (Figure 3). There was no sex predilection (53.3% female). The median lesion duration was 6 months (range: 3 weeks to 36 months). Symptoms were reported in 30 patients and included easy bleeding in 29 patients and pain in one patient. Current or prior orthodontic treatment was reported in 22 patients (7.6%). Other presumed associated factors were malpositioned teeth (19 patients, 6.6%), mouth breathing (one patient), denture wear (one patient), and previous trauma (one patient).

**Figure 4:**
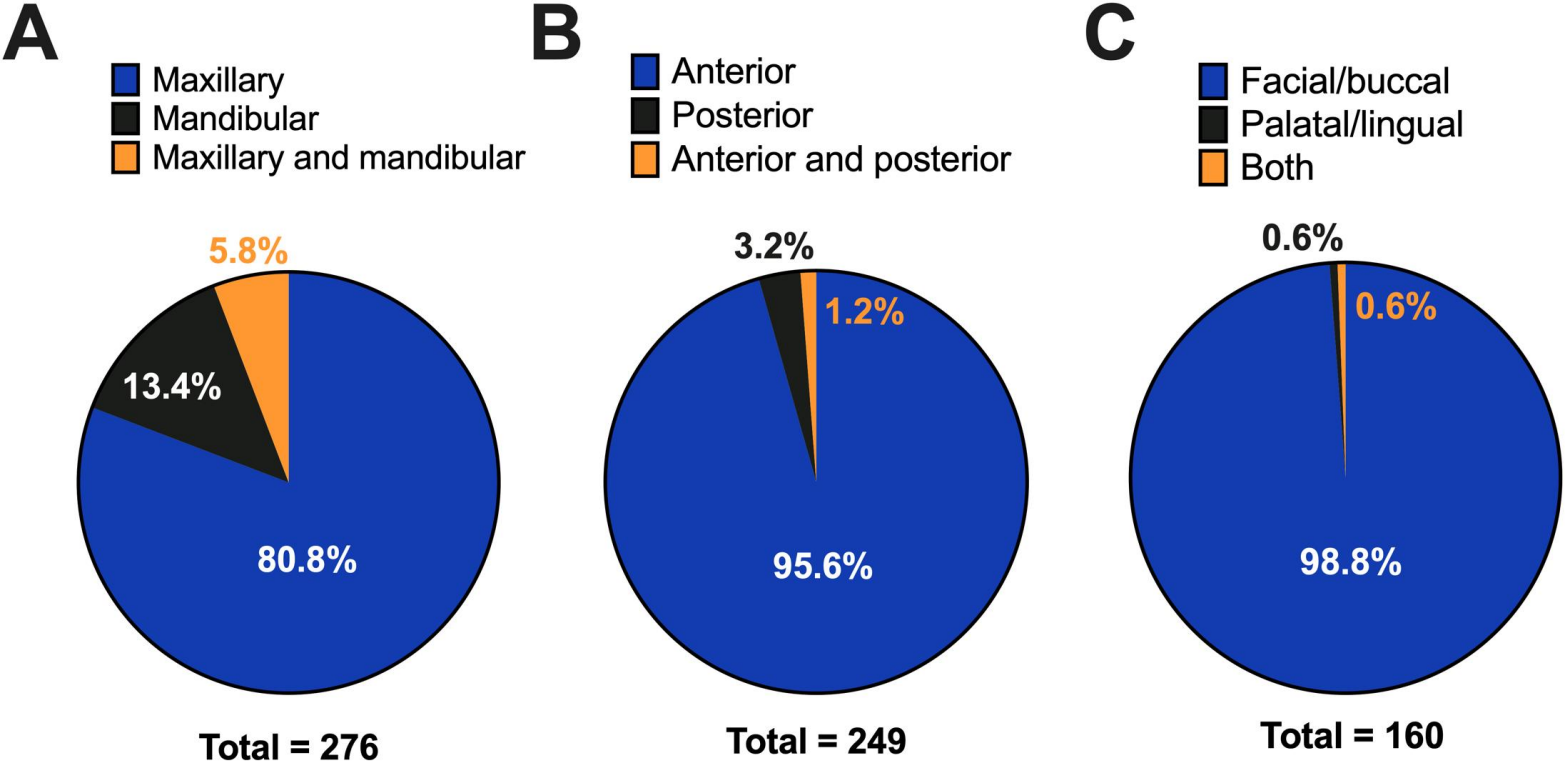
Site distribution of spongiotic gingival hyperplasia based on jaw mucosa affected **(A)**, jaw region **(B)**, and surface **(C)**.

The majority of patients (86.5%) had solitary lesions. Furthermore, 39 patients had more than one SGH lesion, and the number of lesions was reported for 12 patients (median 2 lesions per patient, range 2–4). The lesion location was known for 279 patients. SGH lesions occurred on the gingiva in 277 patients (99.3%); the remaining two patients had lesions on the palatal mucosa (one patient with a single lesion) and alveolar ridge mucosa (one patient with four lesions). Predilections for the maxillary gingiva (80.8%, Figure 4A), anterior region (95.6%, Figure 4B), and facial/buccal area (98.8%, Figure 4C) were noted.

### Histopathologic features

Table 1 summarizes the histopathologic features described in the literature from the 292 biopsies from 289 patients. Surface elevation was noted in 38.4% of biopsies, and more than half (62.7%) had a papillary or corrugated surface. The epithelium was non-keratinized stratified squamous in the majority of cases (73.6%) and was frequently acanthotic/hyperplastic (88.4%). Epithelial atrophy or normal epithelial thickness was rare (1.4% and 1.0%, respectively), whereas atrophy of the suprapapillary plate was not uncommon, occurring at a rate of 17.8%. Spongiosis was a characteristic finding and was reported in 93.8% of the biopsies. Rete ridges were mostly elongated. Although neutrophils were the most commonly transmigrated cells (62.0%), inflammation within the lamina propria was more commonly mixed (48.6%) or chronic (34.2%) compared to purely acute inflammation (0.7%). A rich vascularized lamina propria was a frequent finding (80.1%).

**Table 1:** Summary of the histopathologic features described in the literature (*n* = 292).

Feature	*N* (%)
Exophytic/elevated surface	112 (38.4)
Papillary/corrugated surface	183 (62.7)
Keratinization	
Non-keratinized	215 (73.6)
Non-keratinized or parakeratinized	21 (7.2)
Parakeratinized	10 (3.4)
Epithelial thickness	
Acanthotic/hyperplastic	258 (88.4)
Atrophy of the suprapapillary plate	52 (17.8)
Pseudoepitheliomatous hyperplasia	8 (2.7)
Atrophic	4 (1.4)
Normal thickness	3 (1.0)
Spongiosis	274 (93.8)
Transmigration	
Neutrophilic	181 (62.0)
Acute and chronic cells	61 (20.9)
Present but type unspecified	26 (8.9)
Lymphocytic	6 (2.1)
Rete ridges	
Elongated	128 (43.8)
Broad	52 (17.8)
Inflammation in the lamina propria	
Acute and chronic inflammation	142 (48.6)
Chronic inflammation	100 (34.2)
Present but type unspecified	5 (1.7)
Acute inflammation	2 (0.7)
No inflammation	1 (0.3)
Vascular lamina propria	234 (80.1)
Less common features (<5%)	
Microabscesses	13 (4.5)
Flat surface	7 (2.4)
Bacterial colonization	5 (1.7)
Edematous lamina propria	4 (1.4)
Intraepithelial hemorrhage	3 (1.0)
Acantholytic cells	3 (1.0)
Intracellular (intrakeratinocyte) edema	2 (0.7)
Dystrophic calcifications	2 (0.7)
Reactive atypia within the basal cell layer	1 (0.3)
Giant cell fibroma-like changes	1 (0.3)

### Immunohistochemistry findings and ancillary testing

Cytokeratin (CK) 19 was the most commonly utilized immunohistochemical marker and was positive in 99.0% of cases in which staining was performed (Table 2). The CK19 staining pattern was described in 99 cases and showed full-thickness positivity throughout the lesional epithelium. Similarly, CK8/18, CK14, and CK5/6 displayed diffuse staining in lesional epithelium. Estrogen (ER) and progesterone receptor (PR) tests were performed in 51 cases (1), all of which were negative. S100 was performed in 10 cases (30) and was negative in all 10. CD20 and CD3 tests were performed in 10 cases (30), which highlighted a greater proportion of CD20+ cells compared to CD3+ cells within the lamina propria of the SGH specimens. The proliferation index was low in 11 cases in which Ki-67 tests were performed.

**Table 2:** Summary of immunohistochemical findings.

Immunohistochemical marker	Positive, *N* (%^a^)	Negative, *N* (%^a^)	Not performed, *N*
Low molecular weight cytokeratins			
CK19	101 (99.0)	1 (1.0)	190
CK8/18	10 (100)	0 (0)	282
CK7^b^	1 (10.0)	9 (90.0)	282
CAM5.2^b^	1 (100.0)	0 (0)	291
High molecular weight cytokeratins			
CK5/6	24 (100)	0 (0)	268
CK4	10 (90.9)	1 (9.1)	281
CK14	10 (90.9)	1 (9.1)	281
CK1/10	10 (100)	0 (0)	282
CK34*β*E12	1 (100)	0 (0)	291

CK, cytokeratin.

^a^ The percentage is based on the sum of positive and negative cases.

^b^ Focal and/or weak staining.

P16 immunohistochemistry (IHC) was performed in 21 cases (8); 47.6% demonstrated high intensity staining, 38.1% demonstrated low intensity staining, and the remaining cases exhibited moderate intensity staining. One of the cases that exhibited strong p16 expression was positive for HPV 31 DNA by polymerase chain reaction. Another study performed HPV IHC in one case, presumably p16, which was negative (40).

### Management and outcomes

**Figure 5:**
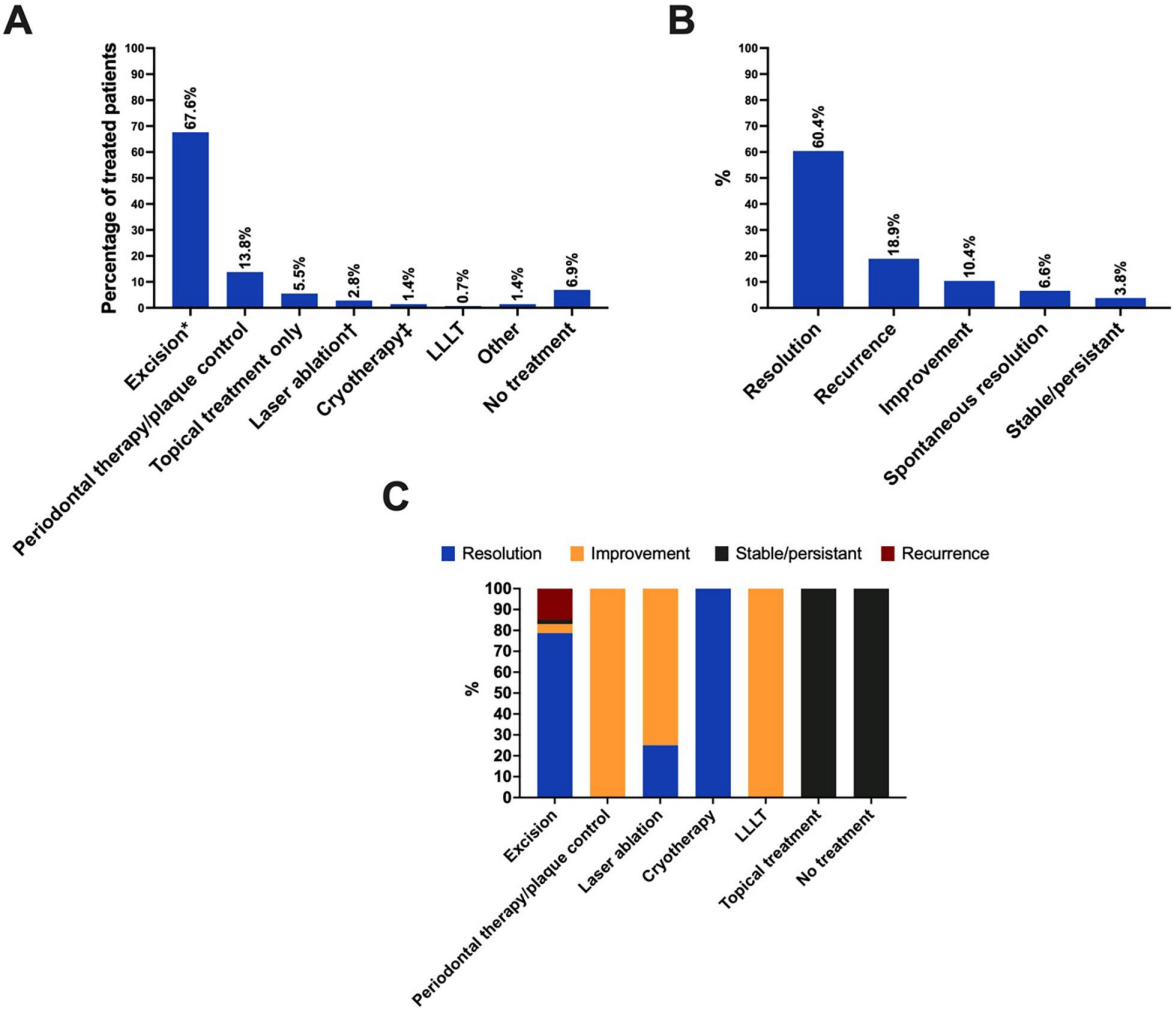
Management and outcomes. **(A)** Spongiotic gingival hyperplasia management. *Of the 98 patients who received surgical excision, five patients received topical treatment following the excision (one for a recurrence and four for unreported reasons). The topical agents consisted of 0.2% chlorhexidine digluconate mouthwash (three patients), 70% trichloroacetic acid solution (one patient), and 0.05% chlorhexidine gel (one patient). In addition, two patients received periodontal care prior to the excision (lesions excised due to lack of response to periodontal therapy). ^†^Of the four patients who underwent laser ablation, one patient received topical clobetasol propionate 0.05% treatment (formulation not specified) before the laser ablation and another patient received topical clobetasol propionate 0.05% ointment following laser ablation. ^‡^One patient had cryotherapy only and another patient underwent cryotherapy following basic periodontal therapy. LLLT, low-level laser therapy. **(B)** Outcomes. **(C)** Outcomes matched with management approaches.

Management was reported for 145 patients. The majority of patients underwent surgical excision (67.6%, Figure 5A). Periodontal therapy was the second most commonly employed treatment (13.8%). Topical treatment alone was used in 5.5% of patients and consisted of trichloroacetic acid [six patients (4)], chlorhexidine gluconate 0.12% rinse [one patient (34)], and topical clobetasol propionate 0.05% [one patient (39)].

The follow-up period was reported for 63 patients and ranged from 1 month to 18 years (median, 12 months). Outcomes were reported for 106 patients, and resolution was documented in 60.4% of patients (Figure 5B). Recurrence was observed in 18.9% of patients overall. The follow-up duration was known for nine of the 20 cases that recurred (median 20 months, range 2–126 months). Furthermore, 62 patients had matched data for management approaches and outcomes. Resolution was documented in 78.7% of patients who underwent excision, and recurrence occurred in 14.9% of patients who underwent excision (Figure 5C).

### Risk of bias

The results of the risk-of-bias assessment are presented in Table 3. The majority of studies (84.0%) had low risk of bias, two publications had moderate risk of bias, and two studies had high risk of bias (Table 3).

**Table 3:** Risk of bias assessment.

Publication	Risk of bias
Case reports	
Damm (33)	Moderate
Flaitz and Longoria (34)	Low
McNeill et al. (35)	Low
Solomon et al. (7)^a^	Low
Nogueira et al. (9)^a^	Low
Moine and Gilligan (13)	Low
Vieira et al. (36)	Low
Roberts et al. (12)	Low
Silveira et al. (5)^a^	Low
Aktypi-Bampouranou et al. (37)	Low
Goldberg et al. (32)^a^	Low
Kala and Prasad (38)	Low
Medeiros et al. (39)	Low
Sidhu et al. (40)	Low
Case series	
Darling et al. (1)	Low
Chang et al. (2)	Low
Argyris et al. (8)	Low
Petrutiu et al. (29)	Low
Lafuente-Ibanez de Mendoza et al. (30)	High
Vargo and Bilodeau. (3)	Low
Wang and Jordan. (16)	Low
Innocentini et al. (31)	High
Theofilou et al. (6)	Low
Gilligan et al. (4)	Low
Case-control study	
Allon et al. (17)	Moderate

^a^ These articles were evaluated using the case reports assessment tool despite having more than one case since the cases were described individually.

## Discussion

In this systematic review, a comprehensive summary of SGH lesions is provided with a focus on the histopathologic features. SGH was described fairly recently in 2007 by Darling and colleagues as juvenile spongiotic gingivitis based on overlapping histopathologic features with spongiotic dermatitis (1). The terminology was shortly changed to localized juvenile SGH by Chang and colleagues (2). The etiopathogenesis of SGH remains uncertain, but it has been attributed to overgrowth of the junctional epithelium, which is the widely accepted hypothesis (17).

SGH tends to occur in individuals younger than the age of 20 (4); hence, the descriptive term “juvenile” was long used in the condition's nomenclature. In the original description of SGH, all but one patient were younger than 20 years (1). In subsequent reports, SGH occurring in individuals older than 20 years of age was identified (3, 6). In this systematic review, SGH occurred most commonly in the first and second decades, with only 6.6% of cases occurring after the second decade.

The classic clinical presentation of SGH is a red plaque, typically with a granular or papillary surface, on the gingiva of the maxillary anterior teeth, and pain is unusual (41). In this systematic review, pain was reported by only one patient (3). In a series of 52 patients with SGH, Chang et al. (2) found a predilection for the maxillary gingiva (83.7%) compared to the mandibular gingiva (16.3%) (2). Similarly, in this systematic review of 289 patients, involvement of the maxillary gingiva was observed in 80.8%. The maxillary predilection was slightly lower (62.9%) in a recent large case series (4). SGH has a predilection for the anterior region (91.7%–100%) compared to the posterior region (1, 2). Indeed, in our study, 95.6% of the cases involved the anterior region. Involvement of the lingual or palatal gingiva is rare and was only reported in two publications (one patient each) (1, 13). One patient in Darling et al. (1) had SGH lingual to teeth #84–85, and the other patient, reported in Moine and Gilligan (13), had SGH on the facial and palatal gingiva (13). SGH has long been known as a gingival lesion, as the name suggests. However, there is one study of two patients with extragingival lesions (5). The first patient had a single lesion on the posterior palatal mucosa, and the second patient had four lesions on the alveolar ridge mucosa (edentulous area). Both patients were older than 50 years of age (5).

SGH exhibits characteristic histopathologic features that are well-documented in a number of case series (1–3, 6, 8, 16). However, the histopathologic features were described narratively in the majority of the aforementioned studies and quantification of such histopathologic features and their variations is lacking. In an attempt to report the histopathologic features of SGH in a quantitative or semiquantitative manner, this systematic review was conducted. This systematic review relied on descriptions of histopathology in the included studies, and it is important to recognize that when a histopathologic feature was not reported in a publication, it does not necessarily indicate that the feature was absent. Hence, the histopathologic features of SGH are likely underrepresented in this systematic review. Theofilou et al. (6) described the histopathologic features of 21 biopsies from 18 patients (6). The majority of cases (66.7%) had a papillary or micropapillary surface on histopathologic examination, which in this systematic review was found in 62.7% of cases. The majority of SGH lesions in Theofilou et al. (6) were covered by non-keratinized stratified squamous epithelium (57.1%), while parakeratinization was seen in 42.9% of cases (6). Parakeratin was reported in a minority of cases in this systematic review. The epithelium of SGH is typically spongiotic and infiltrated by inflammatory cells, and the lamina propria is characteristically vascular (1, 3, 4).

Although the histopathologic features of SGH are characteristic and sufficient for diagnosis, immunohistochemistry has been used in previous studies primarily for diagnostic confirmation or to elucidate the etiopathogenesis of SGH. In the original description of SGH, Darling et al. (1) concluded that SGH was unrelated to puberty, based on the absence of ER and PR protein expression on immunohistochemistry (1). The authors postulated that SGH represented ectopic junctional epithelium, as evidenced by full-thickness expression of CK19 (1). In healthy junctional epithelium, CK19 is characteristically strongly and diffusely expressed across all epithelial cell layers, whereas in sulcular epithelium and oral gingival epithelium, CK19 is exclusively expressed within the basilar and suprabasilar cells (42, 43). Subsequent reports of SGH found a similar full-thickness CK19 staining pattern (6, 17) and concluded that SGH is likely exteriorized junctional epithelium with reactive changes (spongiosis and inflammation) (17). Pritlove-Carson et al. (43) compared the cytokeratin expression pattern of inflamed gingiva to healthy gingiva. CK19 was expressed in the basal cells of healthy gingival epithelium, and the staining increased in intensity in inflamed gingiva but remained confined to the basal cell layer (43). Thus, the pattern of CK19 expression is helpful in distinguishing SGH from other inflammatory gingival lesions.

In general, overexpression of p16 is suggestive of transcriptionally active high-risk HPV (44). The role of p16 expression in SGH has been previously investigated (8, 40). Argyris et al. (8) investigated the role of HPV in 21 SGH lesions. The majority of cases showed strong p16 staining in more than 50% of epithelial cells. Follow-up HPV DNA testing revealed unequivocal HPV31 positivity in one case, with the remaining cases being negative or having suboptimal DNA levels for complete testing (8). Current guidelines, which were published after Argyris et al.’s (8) findings, consider p16 positivity when immunostaining is present in more than 70% of cells with moderate or severe intensity (45). It is possible that some cases in Argyris et al. (8) would be interpreted as p16 negative in the context of current HPV testing guidelines. It would be interesting to reexamine the role of HPV in SGH, considering the current guidelines. Nonetheless, based on current evidence, it can be concluded that p16 negativity combined with CK19 full-thickness expression can be used to differentiate SGH from histologic mimics.

Traditionally, SGH was managed by excision (1, 7). Other methods proposed in the literature include laser ablation, cryotherapy, low-level laser therapy, and topical medications (4, 9, 12, 32, 36). In this systematic review, complete resolution of SGH was noted in lesions treated by excision, laser ablation, and cryotherapy; the remaining methods achieved at best some level of improvement. Nonetheless, the limited response to other methods was based on a limited number of cases published in case reports and case series. Studies with larger sample sizes are needed to understand SGH's response to alternative methods. Recurrence is not uncommon following excision, and the rate was found to be 18.9% overall and 14.9% for excision in this systematic review. Although the follow-up duration was known for a few recurrent cases, the median follow-up duration of the recurrent cases was longer than the overall follow-up duration in this study (20 months vs. 12 months, respectively), suggesting that SGH lesions may exhibit late recurrences. Therefore, long-term follow-up for more than 1 year is recommended. Interestingly, a recent case series observed spontaneous resolution in 10% of cases (4).

This systematic review was limited to publications with biopsy-proven SGH. Therefore, the clinical findings, management, and outcomes may be incomplete because lesions diagnosed solely on clinical impression were excluded. The lack of individual patient data on age (e.g., when mean age was reported) may have introduced bias; however, the median age and age distribution in our analysis were consistent with previous research (2, 4). The small number of multifocal cases and the lack of individual patient data in all cases precluded a direct comparison between unifocal and multifocal lesions. Furthermore, as outlined above, the histopathologic features quantified here were based on the descriptions provided in the publications, which may not have been detailed accounts of all SGH histopathologic features. Consequently, the percentages reported here most likely underestimate the key histopathologic features of SGH. Finally, the small number of cases with reported outcomes and the heterogeneity of the data limit the interpretation of management outcomes.

In conclusion, SGH is an inflammatory/reactive lesion that occurs primarily on the gingiva with a predilection for the maxillary gingiva in the anterior and facial regions. Multifocal lesions were reported in 13.5% of patients included in this systematic review. SGH characteristically presents with an exophytic papillary surface covered by spongiotic non-keratinized stratified squamous epithelium with transmigrated neutrophils and an underlying vascular lamina propria.

## Data Availability

The original contributions presented in the study are included in the article/Supplementary Material, further inquiries can be directed to the corresponding author.
